# Effects of different etching strategies on the microtensile repair bond strength of beautifil II giomer material

**DOI:** 10.4317/jced.54436

**Published:** 2018-08-01

**Authors:** Mahmoud Bahari, Siavash Savadi-Oskoee, Soodabeh Kimyai, Ayda Savadi-Oskoee, Farhang Abbasi

**Affiliations:** 1 Assistant Professor, Dental and Periodontal Research Center and Department of Operative Dentistry, Dental Faculty, Tabriz University of Medical Sciences, Tabriz, Iran; 2 Professor, Department of Operative Dentistry, Dental Faculty, Tabriz University of Medical Sciences, Tabriz, Iran; 3 General Practitioner, Department of Operative Dentistry, Dental Faculty, Tabriz University of Medical Sciences, Tabriz, Iran; 4 Professor, Department of Polymer Engineering, Polymer Engineering Faculty, Sahand University of Technology, Tabriz, Iran

## Abstract

**Background:**

Considering the differences in the filler particles between giomer and conventional composite resins and the importance of these fillers in the repair bond strength, the aim was to evaluate the effects of different etching strategies with phosphoric acid (PA) and hydrofluoric acid (HF) on the microtensile repair bond strength (µTRBS) of giomer.

**Material and Methods:**

Ten giomer blocks were randomly assigned into 10: 1) control; 2) 37%PA-20s; 3) 3%HF-20s; 4) 3%HF-120s; 5) 9.6%HF-20s; 6) 9.6%HF-120s; 7) 37%PA-20s + 3%HF-120s; 8) 37%PA-20s + 9.6%HF-120s; 9) 3%HF-120s + 37%PA-20s; 10) 9.6%HF-120s + 37%PA-20s. In all groups, the One-Step Plus bonding system was applied and the new giomer block was bonded to the existing giomer. After cross-sectional cutting, 18 samples were prepared from each block and the µTRBS of the samples was measured at a strain rate of 0.5 mm/min. Data were analyzed with one-way ANOVA and post hoc Tukey tests (*P*<0.05).

**Results:**

The µTRBS in groups 4, 5, 6, 7, 8 and 10 were significantly higher than that in the control group (*P*<0.05). The µTRBS in group 2 was even less than that in the control group (*P*<0.001). The highest µTRBS was recorded in group 10, which was significantly different from those in groups 3, 4 and 9 (*P*<0.05). In addition, the differences between group 9 and groups 6, 7 and 8 were significantly different (*P*<0.05).

**Conclusions:**

Etching with PA resulted in a decrease in µTRBS. Etching with HF, except for 3%HF-20s and HF after etching with PA, resulted in a significant increase in giomer`s µTRBS. An increase in the application time of 3%HF resulted in a significant increase in the µTRBS.

** Key words:**Dental restoration repair, Hydrofluoric acid, Phosphoric acid, etching.

## Introduction

Composite resin restorations may fail in the long term due to various reasons such as caries, fractures, discoloration and improper contour, necessitating replacement or repair. Replacement of the whole restoration is an invasive procedure and due to the tooth-colored nature of composite resins such a procedure might result in a two-fold loss of sound tooth structure and an increase in the risk of pulpal damage compared to the replacement of amalgam or glass-ionomer restorations. Repair of defective composite resin restorations is an alternative conservative procedure and is preferable in terms of its cost and chair time, in addition to its contribution to the preservation of sound tooth structure and prevention of pulpal damage (1). In the repair of composite resin restorations, a new layer of composite resin is placed over the old composite resin restoration. However, such a procedure is associated with some specific problems due to the absence or presence of a very thin layer of carbon double bonds on the surface of old composite resin for bonding with the new composite resin. Furthermore, in many cases, the old composite resin is unknown, increasing the complexity of the repair process.

Since it is important to achieve a reliable and durable bond between the old and new composite resins for clinical success, it is necessary to increase the surface roughness of the old composite resin to increase the mechanical interlocking and apply a proper bonding agent for surface wetting in order to achieve a chemical bond between the two materials that is strong enough. In addition, many attempts have been made to introduce primers specific for composite resins. However, bonding the new composite resin to the old composite resin layers is still a challenge (2).

Various chemical and mechanical surface preparation techniques and different bonding systems have been suggested to improve the repair bond strength of composite resins. Many studies have shown that surface roughening of composite resins is more effective than the application of bonding agents in improving the repair bond strength (3,4). It is possible to create surface roughness on old composite resin restorations through macromechanical roughening with the use of burs and through micromechanical techniques such as acid etching with hydrofluoric acid or sandblasting. One study shown that surface treatment with a diamond bur or sandblasting results in the highest bond strength (2). Etching with hydrofluoric acid is a reliable technique for bonding composite resins to porcelain. However, the results of studies on the effect of etching composite resin on the repair bond strength to composite resin have been very contradictory. Some studies have failed to show the positive effect of hydrofluoric acid on the repair bond strength of composite resin (2,5). However, some other studies have shown the positive effect of this technique (6,7). The discrepancies in the results of different studies might be attributed to differences in the chemical compositions of composite resins that have been repaired. The repair bond strength is affected by the type of resin monomer and the type and amount of filler in the composite resin. The effect of etching predominantly depends on the chemical composition of filler particles of composite resin; therefore, the effect of etching on the surface roughness of different composite resins will be different (8).

Recently a new group of composite resin-based tooth-colored restorative materials have been introduced, referred to as giomers, which are methacrylate-based. These restorative materials have filler content different from that of conventional composite resins. In fact, giomers are a true combination of glass-ionomers and composite resins, in which the acid‒base reaction has taken place before mixing the fillers with resin, contrary to acid-modified composite resins. After this reaction, the material is lathed and mixed with the methacrylate resin as filler. In this type the release of fluoride is preferred and the mechanical properties are scarified for the release of fluoride. *In-vitro* studies showed that micromechanical properties and biocompatibility of giomer are better in comparison to conventional composite resins. In addition, its excellent clinical performance were shown in several short-term studies and a recent long term 13-year recall examination (9,10).

Since the type of the old composite resin restoration and its filler type as a substrate have an important role in the repair bond strength (11,12), the aim was to evaluate the effect of different etching strategies with phosphoric acid (PA) and hydrofluoric acid (HF) on the microtensile repair bond strength (µTRBS) of giomers. The null hypothesis was that different etching strategies with phosphoric acid (PA) and hydrofluoric acid (HF) have similar effects on the microtensile repair bond strength (µTRBS) of giomers.

## Material and Methods

-Preparation of Samples

First two polylactic acid molds (PLA Shanghai, Guanghe Bio-Tech Co.), measuring 5×20×5 mm were prepared in order to prepare giomer samples with the same size. Beautifil II giomer (Shofu Dental Corporation, Osaka, Japan) was placed in the molds using the incremental technique in 2-mm layers and each layer was light-cured using Demetron A2 (Kerr, West Collins, Orange, CA, USA) light-curing unit at a light intensity of 1000 mW/cm2 for 20 seconds. A glass slab was placed on the last layer before light-curing and after removal of the excess material it was light-cured with the same light-curing unit. Then the giomer blocks were polished with the use of 600-, 800- and 1200-grit silicone paper using the ultrasound technique for 10 minutes.

-Surface preparation 

The prepared blocks were subjected to a 500-round thermocycling procedure in a water bath at 5‒55±5ºC, with a dwell time of 30 seconds and a transfer time of 10 seconds. Then the giomer blocks were randomly assigned to 10 groups and their polished surfaces were prepared using one of the following techniques.

1) Control (no surface treatment)

2) Etching with 37% PA for 20 seconds (37PA-20S)

3) Etching with 3% HF for 20 seconds (3HF-20S)

4) Etching with 3% HF for 120 seconds (3HF -120S)

5) Etching with 9.6% HF for 20 seconds (9.6HF-20S)

6) Etching with 9.6% HF for 120 seconds (9.6HF-120S)

7) Etching with 37% PA for 20 seconds followed by etching with 3% HF for 120 seconds (37PA-20S+3HF-120S)

8) Etching with 37% PA for 20 seconds followed by etching with 9.6% HF for 120 seconds (37PA-20S+9.6HF-120S)

9) Etching with 3% HF for 120 seconds followed by etching with 37% PA for 120 seconds (3HF-120S+37PA-20S)

10) Etching with 9.6% HF for 120 seconds followed by etching with 37% PA for 20 seconds (9.6HF-120S+37PA-20S)

-Repair process

After surface treatments, the surface of each prepared resin block was covered with a thin layer of One Step Plus (Bisco, Schaumburg, USA) adhesive system, followed by thinning with an air stream from an air syringe and light-curing with the same light-curing unit for 10 seconds according to manufacturer’s instructions. Then the new giomer, as a repair material, was placed incrementally in 2-mm layers within a transparent mold and each layer was light-cured with Demetron A2 light-curing unit at a light intensity of 1000 mW/cm2 for 20 seconds. Before light-curing the last layer, the samples prepared in the previous stage were placed on this sample and light-cured for 20 seconds from each side after elimination of excess material in order to achieve a block that measured 10×20×5 mm. Then the interface underwent a 500-round thermocycling procedure in a water bath at 5‒55±5ºC.

-Microtensile repair bond strength test

**Figure 1:**
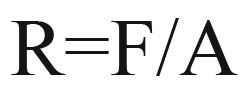
Formula.

The prepared blocks were sectioned with the use of a diamond disk at low speed under water spray. To this end, first the blocks were fixed on the metal rod of the sectioning machine with the use of cyanoacrylate adhesive gel, at a right angle to the diamond disk of the cutting machine. The first section, measuring almost 0.5 mm, was discarded due to the possibility of a deficiency in the adhesive material at the interface, which might have changed the results. Each segment was turned 90º and fixed on the metal rod again. The first section was discarded again for the reason mentioned above. This way, 18 samples were achieved from each giomer block, with an approximate surface area of 1 mm2. Samples with a free adhesive surface were fixed on the special cylinder of the microtensile tester (Bisco, Schaumburg, USA) with the use of cyanoacrylace gel. Microtensile bond strength test was carried out at a strain rate of 0.5 mm/min. The bond strength was calculated using the following formula, (Fig. 1):

Where R is the bond strength in MPa, F is the force required to detach the segments in Newton (N) and A is the bonding surface area in mm2, which was measured with a digital Vernier before the test.

Evaluation of microstructure and surface topography 

In order to evaluate microstructure and the surface topography after the application of different etching strategies, two extra samples of giomer from each group were prepared without the use of any bonding system and new giomer on their surface. The surface topography was evaluated under a Nanoscope® II atomic force microscope (AFM) (Digital Instruments, USA) and the microstructure was evaluated under a scanning electron microscope (SEM) (Cam Scan MV2300, Czech Republic). To prepare the samples for evaluation under an electron microscope, the sample surfaces were gold-sputtered at an angstrom level in the presence of argon plasma. Then the microstructure was photomicrographed under the electron microscope based on the information achieved from the bounced electrons. It should be pointed out that no surface preparation is necessary for evaluation and imaging of the samples under AFM and the samples can be evaluated and photomicrograph under AFM, directly.

-Analysis of data

Normality of data distribution was checked with the Kolmogorov-Smirnov test. One-way ANOVA was used to compare the µTRBS, and post hoc Tukey tests were used for two-by-two comparisons of the groups. Statistical significance was set at *P*<0.05.

## Results

 Table 1 present the means and standard deviations of µTRBS values in the study groups. Kolmogorov-Smirnov test showed that data were distributed normally and were parametric (*P*>0.05). The results of one-way ANOVA showed significant differences in mean µTRBS values between the study groups (*P*<0.001).

**Table 1:**
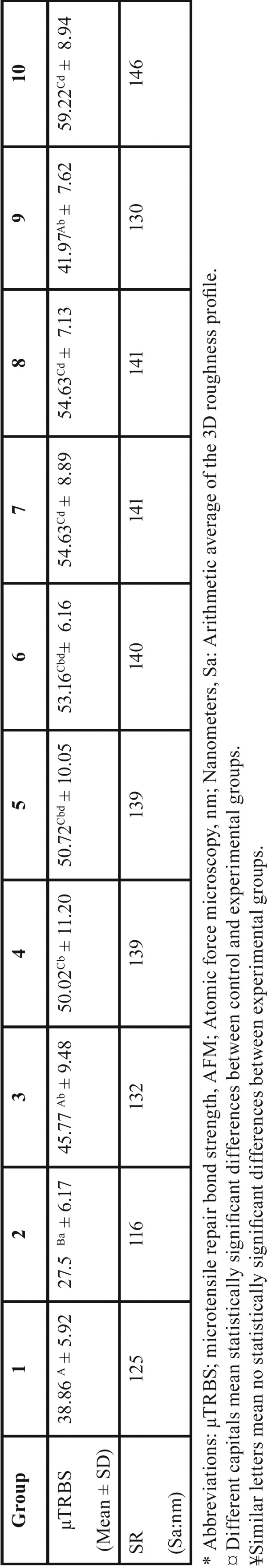
The means and standard deviations (SD) of microtensile repair bond strengths (MPa) and surface roughness (SR) values based on AFM data.

Two-by-two comparisons of the groups with post hoc Tukey test showed that.

1. There were significant differences in the µTRBS of giomer between the control group and all the other groups except for groups 3 and 9; the mean µTRBS in group 1 was higher than that in group 2 and less than that in other study groups (*P*<0.05).

2. There were significant differences in the mean µTRBS of group 2 and all the other study groups; the mean µTRBS in group 2 was less than that in the other study groups (*P*<0.001).

3. There were significant differences in the mean µTRBS of giomer between group 3 and groups 7, 8 and 10, with the least mean in group 3 among these groups (P<0.05).

4. There were significantly differences in the mean µTRBS of groups 6, 7, 8 and 10 and group 9, with the least mean in group 9 among all these groups (*P*<0.05).

5. The highest mean µTRBS of giomer was recorded in group 10, which was significantly higher than those in groups 1, 2, 3, 4 and 9; but it was not significantly higher than those in other study groups.

-Evaluation of microstructure and surface topography 

**Figure 2:**
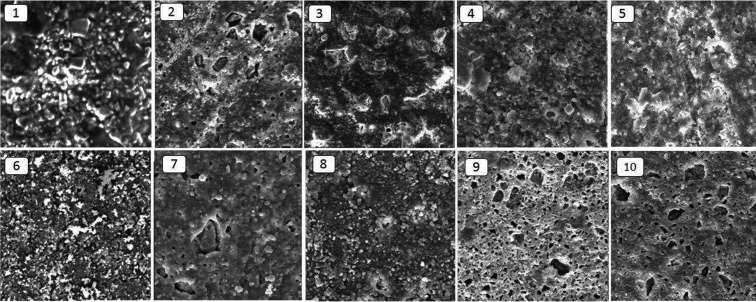
Scanning Election Microscopy photo-micrographs of surface of samples in study groups: 1) Control, 2) 37PA-20S, 3) 3HF-20S, 4) 3HF-120S, 5) 9/6HF-20S, 6) 9/6HF-120S, 7) 37PA-20 S+3HF-120S, 8) 37PA-20 S+9/6HF-120S, 9) 3HF-120S+37PA-20S, 10) 9/6HF-120S+37PA-20S.

**Figure 3:**
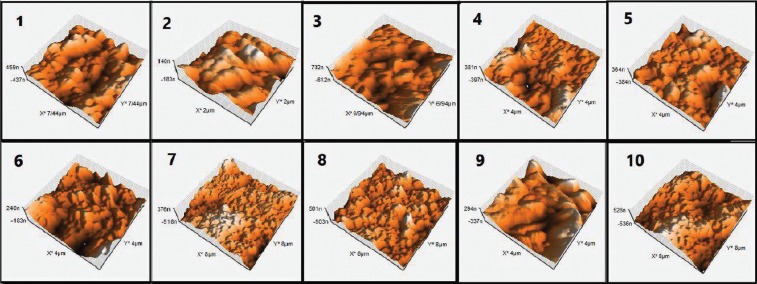
Atomic Force Microscopy photo-micrographs of surface of samples in study groups: 1) Control, 2) 37PA-20S, 3) 3HF-20S, 4) 3HF-120S, 5) 9/6HF-20S, 6) 9/6HF-120S, 7) 37PA-20 S+3HF-120S, 8) 37PA-20 S+9/6HF-120S, 9) 3HF-120S+37PA-20S, 10) 9/6HF-120S+37PA-20S.

Figure 2 presents the SEM photomicrographs for the evaluation of the microstructure of samples after different etching strategies. The results of the evaluation of surface topography under AFM and the relevant numeric values for surface roughness (Sa), are presented in Figure 3 and  Table 1.

## Discussion

Achieving a proper interfacial bond between old and new composite resins and making sure of its durability has always been a challenge due to the absence of the oxygen-inhibited layer, the high conversion rate of monomers and the release of the bulk of monomer with the unreacted double bonds over time (13-17). Since the solubility and penetrability of polymers decrease with an increase in convention rate (18,19), it is very important to create surface roughness and provide conditions for maximum mechanical interlocking during the repair process of old composite resins.

Due to a lack of sufficient data on the proper technique to repair giomers that differ from conventional composite resins structurally and in relation to their filler content, in the present study different techniques were evaluated for creating surface roughness with the use of a single etching process or the combined use of PA and HF to evaluate their effect on the µTRBS of giomers.

The results showed significant increases in µTRBS values compared to the control group after surface preparations, including etching with 9.6% HF for 20 and 120 seconds, etching with 3% HF for 120 seconds, etching with 37% PA for 20 seconds followed by etching with 3% HF for 120 seconds, etching with 37% PA for 20 seconds followed by etching with 9.6% HF for 120 seconds, and etching with 9.6% HF for 120 seconds followed by etching with 37% PA for 20 seconds.

**Figure 4:**
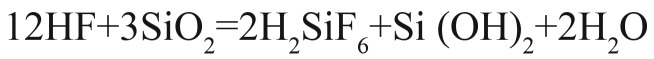
Formula.

Previous studies have shown that the surface roughness of the matrix has a very important role in increasing the bond strength because an increase in surface roughness results in an increase in free surface energy and wettability of adhesive systems (5,20-22). In addition, an increase in surface roughness results in an increase in the possibility of mechanical interlocking and availability of more carbon‒carbon double bonds that are able to form a double bond with their counterparts in the adhesive systems applied on the prepared surfaces for the repair process (23). Therefore, the results above can easily be explained by the AFM images and the surface roughness values resulting from them. However, surface treatment in the form of etching with 3% HF for 20 seconds did not have a significant effect on the µTRBS. A lack of significant increase in surface roughness might be explained by inadequate power of 3% HF to dissolve the superficial giomer fillers and also by its short application time. In contrast, an increase in the duration of application of 3% HF from 20 to 120 seconds resulted in a significant increase in µTRBS (group 4: 3% HF for 120 seconds) compared to the control group. Kula *et al.* (24) showed that an increase in the duration of application of 1.23% APF resulted in greater changes in surface characteristics of composite resins, with greater loss of composite resin weight due to dissolution of a large amount of filler particles, consistent with the results of another study by these researches (25). A study showed that an increase in the duration of application of APF resulted in a significant increase in the weight loss of posterior composite resins with silicate, glass and aluminosilicate filler particles.(24) Silicon dioxide is a glass component which can be dissolved by HF according to the following formula, (Fig. 4):

As a surprise finding, etching with 37% PA for 20 seconds resulted in a significant decrease in bond strength compared to the control groups. As shown by AFM photomicrographs, etching with 37% PA for 20 seconds resulted in erosion and wear and significant smoothening of rough and regular surfaces compared to the control group. This also happened with etching with 3% HF for 120 seconds, followed by etching with phosphoric acid for 120 seconds because as shown by AFM evaluations, the subsequent use of PA resulted in the wear of some surface roughness created by 3% HF, resulting in a decrease in surface roughness.

On the other hand, although surface etching with 37% PA for 20 seconds decreased the surface roughness, subsequent etching with HF, irrespective of the concentration used, resulted in a significant increase in surface roughness, improving the repair bond strength. Comparison of the surface ultrastructure of samples in these groups with the group in which 37% PA was applied for 20 seconds showed that subsequent etching with HF resulted in several surface irregularities in the globular form in the eroded areas resulting from etching with 37% PA for 20 seconds.

Another important findings of the present study was the fact that the µTRBS in 9.6% HF groups for 20 seconds, 9.6% HF group for 120 seconds, and 9.6% HF group for 120- seconds followed by 37% PA for 20 seconds were not significantly different from each other, indicating that increasing the duration of application of 9.6% HF did not result in a significant increase in surface roughness and in bond strength. In addition, if the first etching procedure of the giomer surface is carried out with HF, there is no need to re-etch the surface with 37% PA. Considering the SEM and AFM photomicrographs, it might be concluded that although the subsequent etching with 37% PA after initial etching with HF resulted in changes in the surface ultrastructure and emergence of further depressions and pits on the surface, it did not result in a significant increase in bond strength and surface roughness.

Compared to a study by Loomans *et al.* (8) on the effect of different etching techniques with HF and PA on the surface characteristics and roughness of hybrid and nano-hybrid composite resins, despite some similarities, there are some significant differences in relation to the effect of etching techniques on the microstructure of these composite resins compared to giomer. In contrast to the results reported by Loomans *et al.*, indicating a significant increase in the repair bond strength of hybrid composite resins after etching with 37% PA for 20 seconds followed by etching with 3% HF for 20 seconds despite the increase in surface roughness, in the present study etching the giomer surface with 37% PA resulted in surface erosion and a decrease in repair bond strength compared to the control group. In addition, etching of giomer surface with 3% HF for 20 seconds did not result in a significant change in the µTRBS of giomer.

In addition, in the study above, there was no significant change in the surface roughness and repair bond strength of nano-hybrid composite resins after etching with 37 % PA for 20 seconds and 3% HF for 20 seconds, somewhat consistent with the results of the present study on giomer. On the other hand, similar to the present study, etching of nano-hybrid composite resin with 3% HF for 120 seconds and also a higher concentration of HF alone or in combination with PA resulted in complete detaching of cluster nano-fillers from the surface of composite resin, resulting in an increase in repair bond strength. Comparison of SEM photomicrographs between these two studies shows that it appears pre-reacted GI fillers in giomer are more resistant to complete dissolution or detachment from the composite resin surface in different acid etching strategies compared to different types of barium glass and colloid silica fillers in hybrid composite resins and zirconia-silica fillers in nano-composite resins; in addition, with an increase in the concentration of acid and elimination of acid application the GI fillers undergo more dissolution compared to their environment, decrease in size and are not completely detached from the composite resin surface. Complete detachment was observed only when a combination of PA and HF, respectively, was used. In addition, when first HF was used, followed by application of PA, the fillers were not completely detached from the composite resin surface, similar to that in nano-hybrid composite resins.

It is obvious that the presence of a large number of separate spherical and cylindrical filler particles with small sizes results in a large surface area for bonding with adhesive systems and repair composite resins compared to larger and multi-surfaced particles that are attached to each other. Since the repair bond strengths in groups 6, 7, 8 and 10 were higher compared to groups 1, 2, 3, 4, 5 and 9, the results of this study are consistent with SEM observations.

An important finding in SEM evaluations was the complete detachment of filler particles from the giomer surface in samples in group 9, which exhibited bond strength values comparable to those in the control group, despite the presence of a large number of porosities, i.e. etching with 3% HF for 20 seconds followed by etching with 37% PA for 20 seconds did not significantly increase the bond strength compared to the control group. This is possibly due to the fact that the presence of a balance between the surface roughness created by etching and the remaining filler on the composite resin with a strong bond with the resin matrix is necessary for achieving sufficient bond strength. Complete detachment of a part of fillers, leaving some fillers which are still attached to the resin matrix similar to that in group 10, resulted in a surface that yielded the maximum repair bond strength among all the study groups.

It is obvious that the surface roughness created by etching the surface of old composite resins that are candidates for repair, with different strategies, is mainly influenced by its chemical composition and the chemical composition of its fillers in particular; therefore, there are many discrepancies between the results of different studies on the effect of etching with different strategies on the repair bond strength of old composite resins. The problem becomes more complicated when the chemical composition and the type of the resin of the composite resin to be repaired are unknown and this is the case in many situations. On the other hand, since the surface roughness is not the only factor involved in determining the bond strength, it is not possible at present to introduce an effective repair strategy for all types of composite resins that are available.

It should be pointed out based on the results above that the present study was carried out *in vitro* and with the use of one adhesive system; therefore, its results cannot be completely extended to clinical situations. In addition, it is possible that use of other adhesive systems with the use of different types of repair composite resins will yield different results.

## Conclusions

It can be concluded under the limitations of the present study that.

1. Etching with PA only for 20 seconds resulted in a significant decrease in µTRBS of giomer.

2. Etching with 9.6% HF, irrespective of the duration of etching, resulted in a significant increase in giomer`s µTRBS, and it seems it is not necessary to increase the duration of etching from 20 to 120 seconds.

3. In relation to 3% HF, an increase in the duration of etching from 20 to 120 seconds resulted in a significant increase in giomer µTRBS.

4. Etching with PA, followed by etching with HF, irrespective of the concentration of HF, did not result in a significant increase in giomer µTRBS, compared to etching with HF only.

5. Etching with HF, followed by etching with PA, resulted in a significant increase in repair bond strength compared to the control group, only when higher concentration of HF was used. In addition, etching with lower concentration did not improve the repair bond strength significantly.

## References

[1] Baur V, Ilie N (2013). Repair of dental resin-based composites. Clin Oral Investig.

[2] Brosh T, Pilo R, Bichacho N, Blutstein R (1997). Effect of combinations of surface treatments and bonding agents on the bond strength of repaired composites. J Prosthet Dent.

[3] Swift EJ Jr, LeValley BD, Boyer DB (1992). Evaluation of new methods for composite repair. Dent Mater.

[4] Kupiec KA, Barkmeier WW (1996). Laboratory evaluation of surface treatments for composite repair. Oper Dent.

[5] Lucena-Martín C, González-López S, de Mondelo JMN R (2001). The effect of various surface treatments and bonding agents on the repaired strength of heat-treated composites. J Prosthet Dent.

[6] Shahdad SA, Kennedy JG (1998). Bond strength of repaired anterior composite resins: an in vitro study. J Dent.

[7] Rinastiti M, Özcan M, Siswomihardjo W, Busscher HJ (2010). Immediate repair bond strengths of microhybrid, nanohybrid and nanofilled composites after different surface treatments. J Dent.

[8] Loomans BA, Cardoso MV, Opdam NJ, Roeters FJ, De Munck J, Huysmans MC (2011). Surface roughness of etched composite resin in light of composite repair. J Dent.

[9] Ikemura K, Pashley DH (2008). A review of chemical-approach and ultramorphological studies on the development of fluoride-releasing dental adhesives comprising new pre-reacted glass ionomer (PRG) fillers. Dent Mater J.

[10] Gordan VV, Blaser PK, Watson RE, Mjor IA, McEdward DL, Sensi LG (2014). A clinical evaluation of a giomer restorative system containing surface prereacted glass ionomer filler: results from a 13-year recall examination. J Am Dent Assoc.

[11] Joulaei M, Bahari M, Ahmadi A, Savadi Oskoee S (2012). Effect of Different Surface Treatments on Repair Micro-shear Bond Strength of Silica- and Zirconia-filled Composite Resins. J Dent Res Dent Clin Dent Prospects.

[12] Bacchi A, Consani RL, Sinhoreti MA, Feitosa VP, Cavalcante LM, Pfeifer CS (2013). Repair bond strength in aged methacrylate-and silorane-based composites. J Adhes Dent.

[13] Sideridou ID, Achilias DS (2005). Elution study of unreacted Bis-GMA, TEGDMA, UDMA, and Bis-EMA from light-cured dental resins and resin composites using HPLC. J Biomed Mater Res B Appl Biomater.

[14] Ortengren U, Wellendorf H, Karlsson S, Ruyter IE (2001). Water sorption and solubility of dental composites and identification of monomers released in an aqueous environment. J Oral Rehabil.

[15] Van Landuyt KL, Nawrot T, Geebelen B, De Munck J, Snauwaert J, Yoshihara K (2011). How much do resin-based dental materials release? A meta-analytical approach. Dent Mater.

[16] Ruyter IE, Svendsen SA (1978). Remaining methacrylate groups in composite restorative materials. Acta Odontol Scand.

[17] Vankerckhoven H, Lambrechts P, van Beylen M, Davidson CL, Vanherle G (1982). Unreacted methacrylate groups on the surfaces of composite resins. J Dent Res.

[18] Takahashi T (1990). [Visible light cured resin. Change of quantity of remaining double bonds in the surface low conversion layer by FT-IR method]. Shika Zairyo Kikai.

[19] Chung KH, Greener EH (1990). Correlation between degree of conversion, filler concentration and mechanical properties of posterior composite resins. J Oral Rehabil.

[20] Cavalcanti AN, De Lima AF, Peris AR, Mitsui FH, Marchi GM (2007). Effect of surface treatments and bonding agents on the bond strength of repaired composites. J Esthet Restor Dent.

[21] Bonstein T, Garlapo D, Donarummo J Jr, Bush PJ (2005). Evaluation of varied repair protocols applied to aged composite resin. J Adhes Dent.

[22] Cho SD, Rajitrangson P, Matis BA, Platt JA (2013). Effect of Er,Cr:YSGG laser, air abrasion, and silane application on repaired shear bond strength of composites. Oper Dent.

[23] Nilsson E, Alaeddin S, Karlsson S, Milleding P, Wennerberg A (2000). Factors affecting the shear bond strength of bonded composite inlays. Int J Prosthodont.

[24] Kula K, Webb EL, Kula TJ (1996). Effect of 1- and 4-minute treatments of topical fluorides on a composite resin. Pediatr Dent.

[25] Kula K, McKinney JE, Kula TJ (1997). Effects of daily topical fluoride gels on resin composite degradation and wear. Dent Mater.

